# Knockdown of LncRNA-H19 Ameliorates Kidney Fibrosis in Diabetic Mice by Suppressing miR-29a-Mediated EndMT

**DOI:** 10.3389/fphar.2020.586895

**Published:** 2020-11-26

**Authors:** Sen Shi, Li Song, Hao Yu, Songlin Feng, Jianhua He, Yong Liu, Yanzheng He

**Affiliations:** ^1^ Department of Vascular Surgery, The Affiliated Hospital of Southwest Medical University, Luhzou, China; ^2^ Key Laboratory of Medical Electrophysiology, Ministry of Education, Collaborative Innovation Center of Prevention and Treatment of Cardiovascular Disease of Sichuan Province, Luzhou, China; ^3^ Cardiovascular and Metabolic Diseases Key Laboratory of Luzhou, Luzhou, China; ^4^ Department of Anesthesiology, The Affiliated Hospital of Southwest Medical University, Luhzou, China; ^5^ Department of Endocrinology, The Affiliated Hospital of Southwest Medical University, Luhzou, China

**Keywords:** TGF-β/SMAD3 singling, kidney fibrosis, long non-coding ribonucleic acid-H19, endothelial-mesenchymal transition, microRNA-29a

## Abstract

Diabetic nephropathy is the leading cause of kidney fibrosis. Recently, altered expressed or dysfunction of some long non-coding RNAs (lncRNAs) has been linked to kidney fibrosis; however, the mechanisms of lncRNAs in kidney fibrosis remain unclear. We have shown that the DPP-4 inhibitor linagliptin can inhibit endothelial-mesenchymal transition (EndMT) and ameliorate diabetic kidney fibrosis associated with DPP-4 protein levels via the induction of miR-29. Here, we found that expression of the lncRNA H19 was significantly up-regulated in TGF-β2-induced fibrosis in human dermal microvascular endothelial cells (HMVECs) *in vitro*, and in kidney fibrosis of streptozotocin-induced diabetic CD-1 mice. We also detected up-regulated H19 expression and down-regulated miR-29a expression in the early and advanced mouse models of diabetic kidney fibrosis. H19 knockdown significantly attenuated kidney fibrosis *in vitro* and *in vivo*, which was associated with the inhibition of the EndMT-associated gene FSP-1. We also found that the up-regulation of H19 observed in fibrotic kidneys associated with the suppression of miR-29a in diabetic mice. H19, miR-29a, and EndMT contribute to a regulatory network involved in kidney fibrosis, and are associated with regulation of the TGF-β/SMAD3 singling pathway. This study indicates that inhibition of LncRNA H19 represents a novel anti-fibrotic treatment for diabetic kidney diseases.

## Introduction

Diabetic nephropathy (DN) is a major cause of morbidity and mortality in patients with both type I and type II diabetes mellitus and is the leading cause of end-stage renal disease worldwide (Loeffler and Wolf, 2015). Kidney fibrosis is usually the final outcome of many renal diseases, of which DN is the leading cause (Kanasaki et al., 2013). Many cellular and molecular events occur in kidney fibrosis such as the activation of interstitial fibroblasts, phenotypic conversion of tubular epithelial and endothelial cells, extracellular matrix (ECM) overproduction, and microvascular dysfunction (Eddy and Neilson, 2006). Our previous studies shown that the endogenous antifibrotic peptide N-acetyl-seryl-aspartyl-lysyl-proline (AcSDKP), the substrate of angiotensin-converting enzyme (ACE), is an orally available peptide drug used to cure kidney fibrosis in diabetic mice. AcSDKP treatment can restore the level of anti fibrosis miRNAs in diabetic mice, such as miR-29s and let-7s (Nitta et al., 2016).

DPP-4 inhibitors have been introduced into the market as antidiabetic drugs. We have found that the DPP-4 inhibitor linagliptin ameliorated kidney fibrosis in diabetic mice without altering the blood glucose levels associated with the inhibition of EndMT and the restoration of microRNA (miR) -29s (Kanasaki et al., 2014). However, whether there are other RNA mechanisms underlying diabetic fibrosis remains largely unclear.

Long non-coding RNAs (lncRNAs) are defined as transcripts longer than 200 nucleotides with little or no protein-coding ability (Ma et al., 2013) and have been reported to participate in a lot of biological and pathological processes such as carcinogenesis and chronic diseases including DN (Briggs et al., 2015; Huarte, 2015; Uchida and Dimmeler, 2015; Li et al., 2018). It has been reported that lncRNAs might function as competing endogenous RNAs (ceRNAs) to regulate the expression of miRNAs (Ma et al., 2013). H19 is a 3 kb lncRNA expressed in the nucleus and cytoplasm and is highly expressed in embryogenesis. The expression of H19 is significantly increased in some diseased conditions (Bartolomei et al., 1991; Matouk et al., 2007; Dudek et al., 2010) and it has been reported to play an important role in renal development (Okamoto et al., 1997).

Xie found that H19 expression was significantly upregulated in TGF-β2-induced HK-2 cell fibrosis and in unilateral ureteral obstruction (Xie et al., 2016). Our preliminary study showed that EndMT and the restoration of miR-29s is associated with TGF-β2-induced kidney fibrosis (Kanasaki et al., 2014). Whether there is a further connection between H19, EndMT, and other signaling pathways remains unclear. Herein, we explored the therapeutic potential and possible mechanisms of H19 in kidney fibrosis in a streptozotocin (STZ) induced diabetic mouse model, examining the mechanism of H19 in kidney fibrosis in association with miR-29a-mediated EndMT.

## Materials and Methods

### Animal Model and Treatment

All animal experimental procedures were approved by the Ethics Committee of the affiliated Hospital of Southwest Medical University. Eight-week-old male CD1 mice (Dossy Laboratory Animal Co. Ltd., Chengdu, China) were administered with a single intraperitoneal injection of streptozotocin (STZ) (200 mg/kg); control mice were injected with citrate buffer. Two weeks after the STZ injection, mice with blood glucose levels >16 mmol/L were confirmed as valid diabetic mice and used for this study. The mice were divided into the following three groups: control, DM and H19 knockdownt group.24 weeks after the initiation of diabetes, the mice were sacrificed. Kidney tissues were isolated and then stored at –80°C for histological, RNA and protein analysis.

### Cell Culture

Human dermal microvascular endothelial cells (HMVECs, Lonza, Basel, Switzerland) were cultured in EGM (Lonza, Basel, Switzerland) containing 10% fetal bovine serum (FBS, Gibco) in a regular CO2 incubator at 37°C under 5% CO2/95% air. When HMVECs reached 70% confluence, they were treated with 5 ng/ml recombinant human TGF-β2 (Abcam, Cambridge, UK) for 48 h to induce fibrosis.

### Transfection

A specific duplex small interfering RNA (siRNA) and a short hairpin RNA (shRNA) against H19, with their respective AAV vectors were synthesized by Vigene Biosciences (Jinan, Shandong, China). CD-1 mice were injected with AAV- shH19 at a dose of 2 × 10^12^ viral genome particles per animal through the tail vein using an insulin syringe and a 30-gauge needle. Mice were sacrificed 4 weeks later. The expression of H19 was analyzed using quantitative real time PCR (qPCR). For *in vitro* transfection studies, HMVECs were passaged in 6-well plates with growth medium; they were then transfected with 100 nM shRNA and an antagomiR against miR-29a using Lipofectamine 2000 transfection reagent (Jinan, Shandong, China), according to the manufacturer’s instructions. HMVECs were transfected with shH19 followed by treatment with TGF-β2 (5 ng/ml) for 48 h to induce fibrosis. The sequences of shH19: GGA​TCC​AGC​AAG​AGC​AGA​A. The sequences of mimetics for miR29s: 29a-3p: UAG​CAC​CAU​CUG​AAA​UCG​GUU​A, 29b-3p: UAG​CAC​CAU​UUG​AAA​UCA​GUG​UU, 29c-3p: UAG​CAC​CAU​UUG​AAA​UCG​GUU​A. The sequences of antagomiR for miR29s: 29a-3p: UAA​CCG​AUU​UCA​GAU​GGU​GCU​A, 29b-3p: AAC​ACU​GAU​UUC​AAA​UGG​UGC​UA, 29c-3p: UAA​CCG​AUU​UCA​AAU​GGU​GCU​A.

### Immunofluorescence

Frozen kidney sections (5 μm) were used for immunofluorescence and the number of double positive cells labeled for FSP-1 (cat. no. ab197896; Abcam) and CD31 (cat. no. ab9498; Abcam) were measured. Briefly, frozen sections were dried and placed in acetone for 10 min at −30°C. Once the sections were dried, they were washed twice in phosphate-buffered saline (PBS) for 5 min and then blocked in 2% bovine serum albumin/PBS for 30 min at room temperature. Thereafter, the sections were incubated in primary antibody (1:400) for 1 h and washed in PBS (5 min) three times. Next, the sections were incubated with the secondary antibodies (1:600) for 30 min, washed with PBS three times (5 min each), and mounted with mounting medium containing DAPI. The immunolabeled sections were analyzed with an Olympus fluorescence microscope (Olympus Corporation, Beijing, China).

### Histology

Mouse kidney specimens were processed for further investigation. The tissues were fixed in 4% paraformaldehyde solution, dehydrated with a series of graded ethanol and embedded in paraffin. Sections (10 μm thick) were stained with hematoxylin and eosin (H&E) and Masson’s trichrome staining (MTS) then photographed under an optical microscope (Leica Imaging Systems, Cambridge, United Kingdom). Masson’s trichrome labeled sections were imaged and analyzed with ImageJ software, and fibrotic areas were quantified.

### RNA Isolation and Quantitative Real Time PCR

Total RNA was extracted from renal tissue or HMVECs using Trizol reagent (Foregene, Chengdu, China). Reverse transcription was performed using the Premix RT EasyTM II (With gDNase) (Foregene, Chengdu, China). All qPCR experiments were performed using SYBR Green real time qPCR Master Mix (Foregene, Chengdu, China) on a Bio-Rad CFX Connect Real Time qPCR Detection system (Bio-Rad Laboratories, Inc.). For the qPCR reactions, two ul cDNA was added to a 20 µl reaction mixture containing 10 µl of 2 × Power SYBR Green qPCR Master Mix with 0.8 µl of each primer. The comparative Ct method was used to detect target gene expression in the test samples relative to control samples. All primers were synthesized by RIBOBIO (Guangzhou, China). 18S RNA level was used as a reference. The primers sequences: H19: 5’-AAG​CAG​ATG​GAA​CAG​GTG​GC-3’ (forward) and 5’-CAC​AGC​CAA​ACT​GCC​CAA​AG-3’ (reverse); miR 29s: miR-29a-3p: 5 -UAG​CAC​CAU​CUG​AAA​UCG​GUU​A, miR-29b-3p: 5i UAG​CAC​CAU​UUG​AAA​UCA​GUG​UU, miR-29c-3p: 5 UAG​CAC​CAU​UUG​AAA​UCG​GUU​A.

### Western Blotting

Protein from renal tissues and HMVECs was extracted using protein lysis buffer (Beyotime Biotechnology Co., Ltd., Shanghai, China). Approximately 20 μg of protein lysates were separated on SDS-PAGE and blotted onto PVDF membranes using semidry transfer. After blocking with 5% BSA/TBST, the membranes were incubated with primary antibodies (1:1000) at 4°C overnight. The membranes were washed thrice by TBST and incubated with secondary antibodies (1:10000) for 1 h at room temperature. The rabbit polyclonal to CD31 antibody (cat.:ab9498; Abcam), rabbit polyclonal to alpha smooth muscle actin (cat: ab5694; Abcam), rabbit polyclonal anti-GAPDH (cat:ab8245; Abcam), rabbit polyclonal anti-TGFβ-receptor I (TGFβ R1) antibody (cat:ab31013; Abcam), rabbit polyclonal anti-TGFβ-receptor-II (TGFβ R2) antibody (cat:ab269279; Abcam), rabbit monoclonal anti-fibroblast specific proteins (FSP1, sometimes displayed as S100A4) antibody (cat:ab197896; Abcam), and rabbit anti-SMAD3 (phospho S423 + S425) antibody (cat:ab40854; Abcam) were purchased from Abcam (Cambridge, UK). The IRDye 800CW goat anti-rabbit IgG secondary antibody (cat:926-32211; LI-COR) was purchased from LI-COR (Nebraska, USA).

### Wound Healing Assay

Wound healing assays were performed to evaluate the migration rate of HMVECs transfected with or without H19 shRNA. HMVECs were placed in six-well plates and using a pipette tip at an angle of 30°, each well received a straight scratch simulating a wound. After 24 and 48 h, the number of cells that had migrated into the wounded area was counted under a light microscope (Leica Imaging Systems, Cambridge, UK).

### Cell Migration Boyden Chamber Assay

The bottom side of the migration chamber (cell culture insert; BD Falcon, San Jose, CA) was coated with Matrigel (BD Biosciences, US), and 1,000 HMVECs were passaged in the upper migration chamber. Twenty-four h after passage, the medium was changed to medium containing the transfection reagents in both the upper and the bottom wells. After 48 h, the cells were washed with PBS, followed by fixation with formaldehyde (3.7% in PBS) at room temperature for 2 min. After washing twice with PBS, the cells were permeabilized with 100% methanol for 20 min at room temperature. Then, cells were washed twice with PBS and stained with H&E. After scraping off the nonmigratory cells (upper well) with a cotton swab, the number of migrated cells was counted under a light microscope (Leica Imaging Systems, Cambridge, UK).

### Assessment of Urinary Albumin and Creatinine Concentrations

Urinary albumin concentration was measured using a Mouse Albumin ELISA quantitation kit (E90-134; Bethyl Laboratories Inc; Montgomery, TX, USA). Assay was conducted according to the manufacturer’s protocol. Urinary creatinine levels were measured using a CREP2 kit (Roche Diagnostics, Meylan, France) according to an established protocol. The urinary albumin to creatinine ratio was calculated.

### Assessment of Serum Creatinine

The concentration of serum creatinine was detected using a creatinine assay kit (cat. no. C011-1; Nanjing Jiancheng Bioengineering Institute). Assay was conducted according to the manufacturer’s protocol.

### Glomerular Filtration Rate

Mice were anesthetized with isoflurane and a miniaturized imager device (Mannheim Pharma and Diagnostics, Mannheim, Germany) was mounted onto the animald) back. The skin background signal was recorded for 5 min before intravenous injection of 150 mg/kg FITC-sinistrin (Mannheim Pharma and Diagnostics, Germany). Then, *trans*-cutaneous fluorescence was recorded for 1 h in conscious animals. GFR (ml/min.g.Kw) was calculated from the decrease in fluorescence intensity over time (ie, plasma half-life of FITC-sinistrin) and an empirical conversion factor using the MPD Lab software (Mannheim Pharma and Diagnostics, Germany). Results are means ± SEM.

### Statistical Analysis

The data are expressed as means ± S.E.M. A one-way ANOVA followed by a Tukey’s multiple comparison test was used to determine significance which was defined as *P* < 0.05, if not otherwise noted. GraphPad Prism software (Ver 7.0) was used for the statistical analysis.

## Results

### H19 Expression Was Significantly Up-Regulated in TGF-β2-Induced HMVEC and in the Fibrotic Kidneys of Streptozotocin-Induced Diabetic CD-1 Mice

**FIGURE 1:**
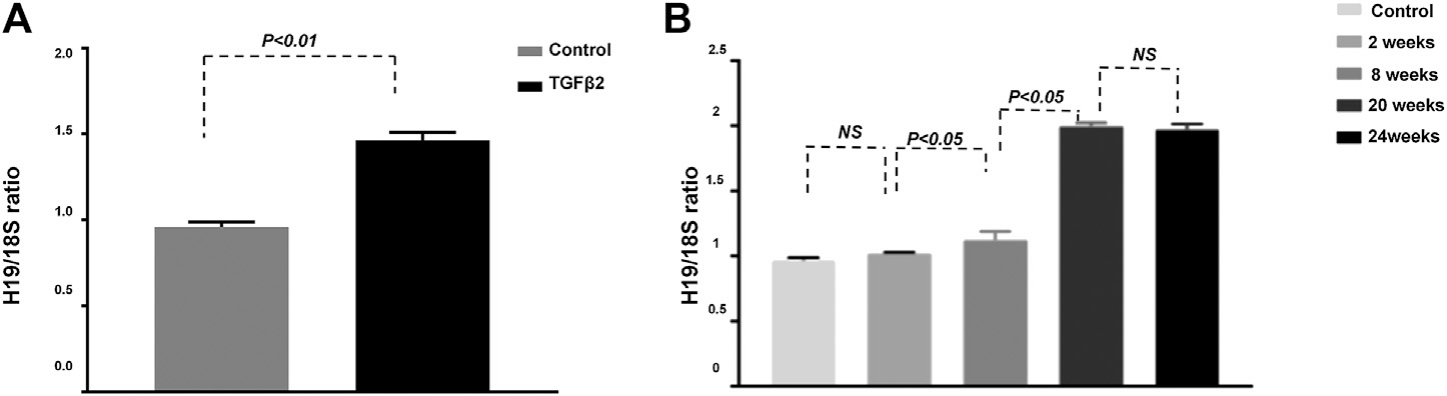
H19 expression is significantly up-regulated in TGF-β2-induced fibrosis *in vitro* and in the kidney of streptozotocin-induced diabetic CD-1 mice. **(A)** qPCR analysis of H19 expression in HMVECs; **(B)** qPCR analysis of H19 kidney expression at different time points after the initiation of diabetes . The data are presented as mean ± SE in each group (n = 5) of three independent experiments.

To determine the pathological significance of H19, we analyzed STZ-induced diabetic male CD-1 mice, a murine model with extensive diabetes-associated kidney fibrosis, and TGF-β2-induced HMVECs (Sugimoto et al., 2007). Our qPCR analysis showed that H19 expression was significantly higher in HMVECs treated with TGF-β2 (Figure 1A). To further investigate the role of H19 in the progression of kidney fibrosis, we analyzed the expression of H19 at different time points after the initiation of diabetes (Figure 1B). We found that in the early period of fibrosis, there was no difference in H19 expression in the kidneys of control and STZ mice; however, after 8 weeks the expression of H19 was significantly higher in the kidneys of STZ mice when compared with control mice, which exhibiting a time dependence. We show that 20 weeks after the initiation of diabetes, the kidneys exhibited serious fibrosis; however, our data showed that the expression of H19 was not different between the 20 and 24 weeks. These data revealed that H19 expression was associated with the progress and severity of kidney fibrosis.

### H19 Knockdown Significantly Attenuated Kidney Fibrosis in the Diabetic Kidney

To further examine the potential relationship between H19 and kidney fibrosis, we treated diabetic mice (DM) with H19 shRNA 20 weeks after the initiation of diabetes and 4 weeks later harvested their kidneys. Our qPCR results confirmed knockdown of H19 in DM treated with shRNA (Figure 1A). We performed H&E and MTS staining to evaluate fibrosis in the kidney. Twenty-four weeks after the initiation of diabetes mice exhibited severe fibrosis when compared with control mice and H19 shRNA-treated DM exhibited restored normal kidney structures (Figure 1B). Our morphometric analysis of the kidneys revealed that DM displayed significantly enlarged glomeruli (Figure 1C), mesangial expansion (D), and relatively large areas of Masson’s trichrome–positive interstitial fibrosis (E), whereas restored normal kidney histology and normal architecture were seen in H19 shRNA treated DM mice. The glomerular functional assays such as Albumin Creatinine ratio (Figure 1F), Glomerular Filtration rate (G) and Serum Creatinine ratio (H) also support the result.

### H19 Knockdown Ameliorated Kidney Fibrosis Was Associated With the Suppression of EndMT

**FIGURE 2:**
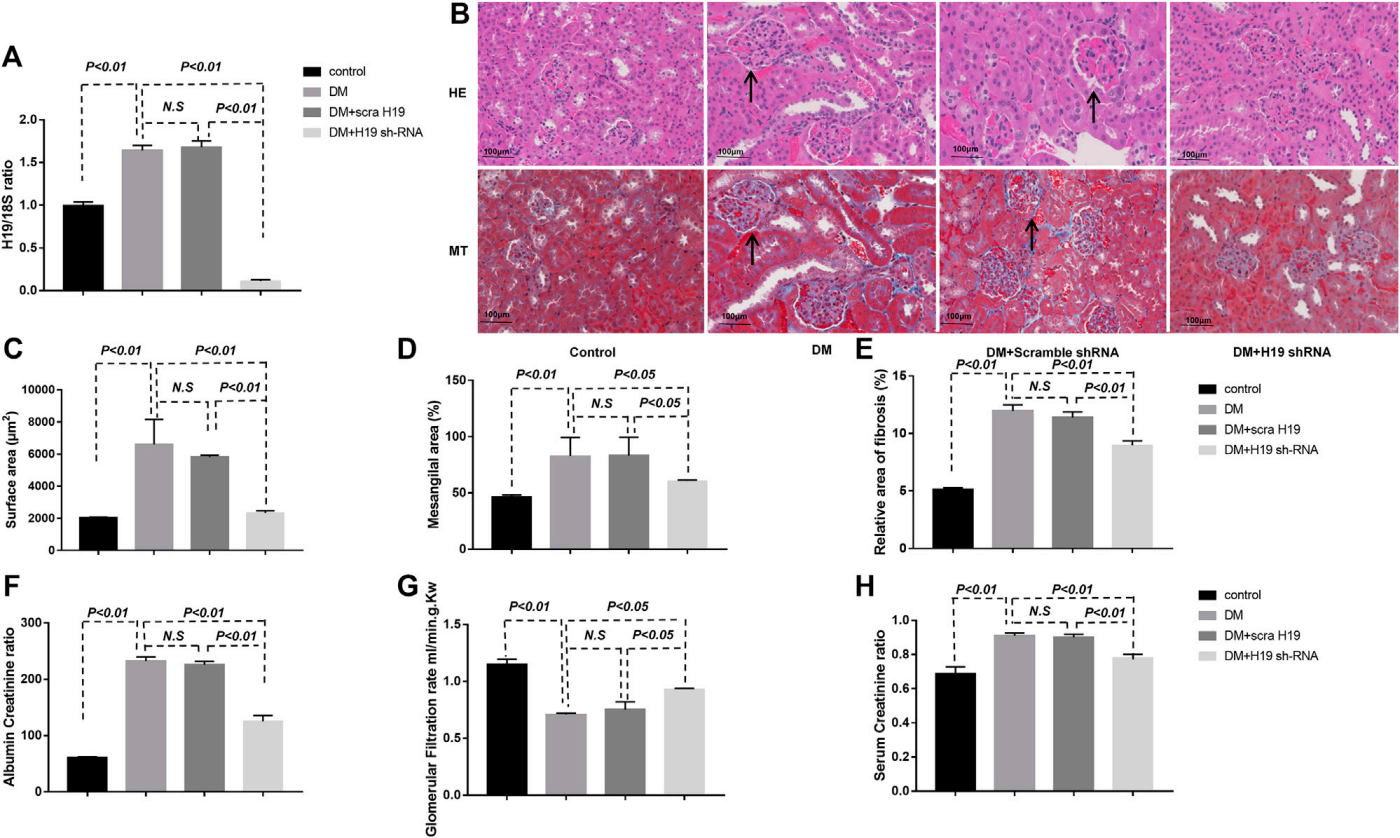
H19 knockdown significantly attenuates fibrosis in the diabetic kidney. **(A)** qPCR analysis of H19 expression in diabetic mice treated with shRNA; **(B)** Representative images of hematoxylin and eosin, and Masson’s trichrome staining used to evaluate fibrosis in the kidney. Scale bar: 100 µm. **(C)**–**(E)** Morphometric analysis of kidney histology. **(F)** Albumin Creatinine ratio, **(G)** Glomerular Filtration rate (ml/min.g.Kw), **(H)** Serum Creatinine ratio. The data are presented as mean ± SE in each group (n = 5) of three independent experiments.

Our previous study showed that EndMT plays an important role in kidney fibrosis (Kanasaki et al., 2014). The inhibition of the EndMT associated gene FSP-1 ameliorated kidney fibrosis *in vivo* and *in vitro*. To confirm the connection between H19 and EndMT, we analyzed EndMT in the kidney of H19 shRNA treated DM mice. Western blot analysis showed the expression of the endothelial marker CD31 was suppressed and the mesothelial cell marker FSP-1 was induced in DM compared with control mice, suggesting the induction of EndMT in the DM kidney; however, when the DM were treated with H19 shRNA, EndMT was repressed (Figure 2A). Immunofluorescence results for FSP-1 (green) and CD31 (red) were in agreement with the western blot data (Figure 2B). Furthermore, we found that TGF- β2 induced EndMT was suppressed by H19 knockdown in HMVECs (Figure 2C). These data revealed that H19 knockdown ameliorated kidney fibrosis is associated with the suppression of EndMT *in vivo* and *in vitro*.

**FIGURE 3:**
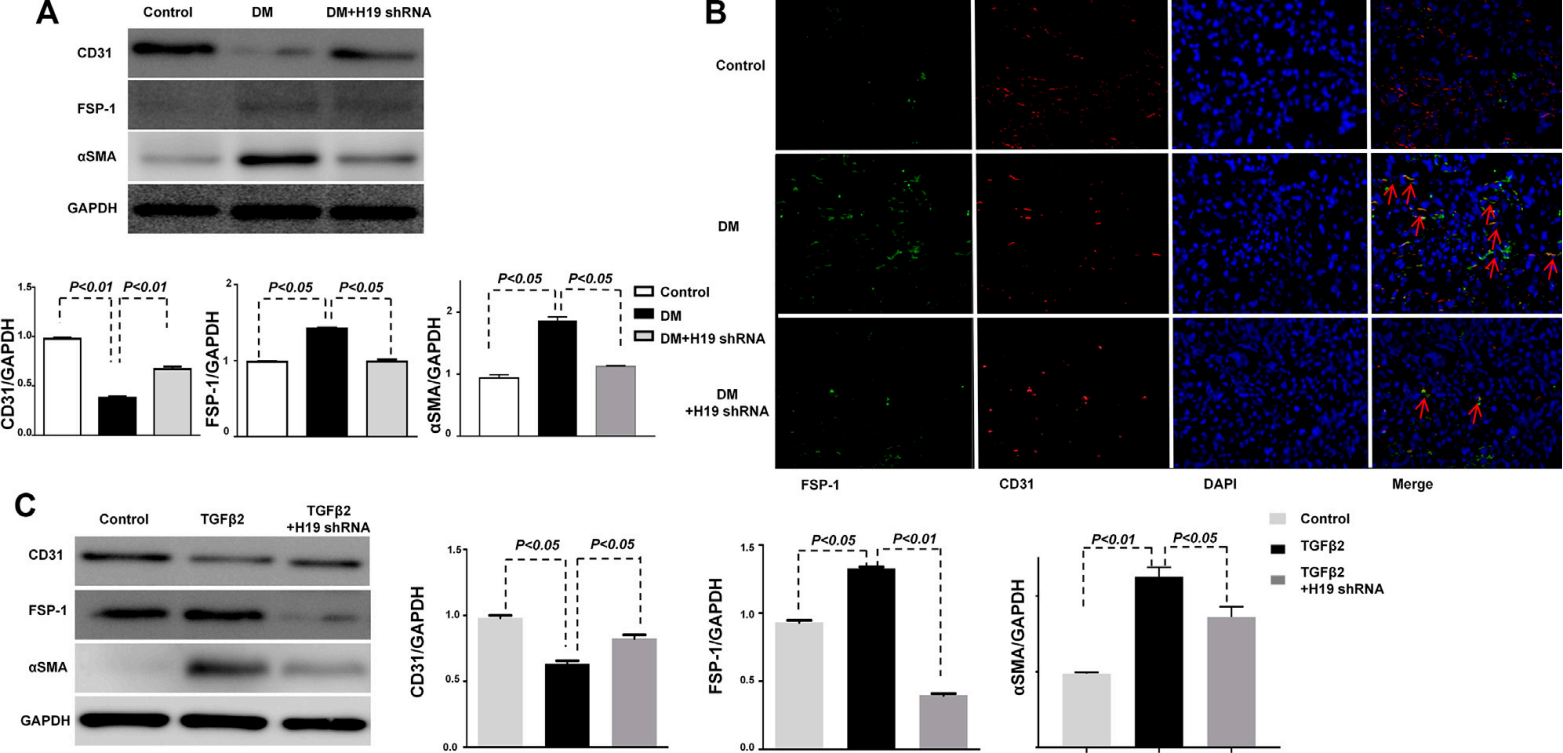
Ameliorated kidney fibrosis by H19 knockdown is associated with the suppression of the EndMT associated gene FSP-1. **(A)** Western blot analysis of CD31, FSP-1, α-SMA, and GAPDH *in vivo*; **(B)** Immunofluorescence analysis of CD31 and FSP-1. The original magnification was ×400. Scale bar: 100 μm in each panel. **(C)** Western blot analysis of CD31, FSP-1, *α*-SMA and GAPDH *in vitro*. The data are presented as meanent in each group (n = 5) of three independent experiments.

We previously showed that EndMT in kidney fibrosis in mediated by miRNA-29 family members (Kanasaki et al., 2014). Whether the protective role of H19 knockdown in kidney fibrosis is related to miRNA-29 family member regulation remains unknown. We therefore confirmed the expression of the miRNA-29 family members *in vivo* and *in vitro* and found that their expression was suppressed in the diabetic kidney, in agreement with our previous research. However, only the expression of miR-29a was restored with H19 shRNA. There was no significant difference in miR-29b and miR-29c expression with or without H19 knockdown (Figures 3A–C). *In vitro*, we also found that TGF-β2 suppressed miRNA-29a could be restored by H19 shRNA in HMVECs, while miR-29b and miR-29c could not (Figures 3D–F). Furthermore, we confirmed the levels of H19 with individual miRNA-29 family member knockdowns; we found that TGF-β2 induced higher H19 expression could only be suppressed with knockdown of miR-29a in HMVECs but not with miR-29b and miR-29c knockdown (Figures 3G–M). These results confirmed that the H19 knockdown mediated kidney fibrosis was associated with miR-29a-mediated EndMT.

### H19 Knockdown Inhibits TGFB/SMAD3 Signaling in the Diabetic Kidneys

**FIGURE 4:**
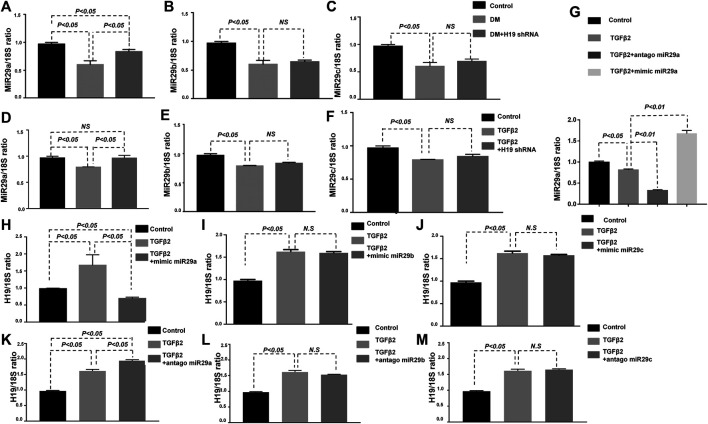
H19 knockdown ameliorated kidney fibrosis was associated with miR-29a. **(A)**–**(C)** qPCR analysis of miRNA 29a/b/c expression *in vivo*; **(D)**–**(F)** qPCR analysis of miRNA 29a/b/c expression *in vitro*; **(G)** qPCR analysis of miRNA 29a expression with diffreent treated. qPCR analysis of lncRNA-H19 expression when **(H)**–**(J)** knockdown miRNA-29a/b/c (the antagonist of miRNA 29a/b/c) and **(K)**–**(M)** over expression miRNA-29a/b/c (the mimic of miRNA 29a/b/c) *in vitro*. The data are presented as meanent in each group (n = 5) of three independent experiments.

Many researches have shown that targeting TGF-β/SMAD3 signaling may represent a specific and effective therapy for kidney fibrosis (Meng et al., 2015; Song et al., 2016). Our research also confirmed that DPP-4 inhibitors ameliorate kidney fibrosis via TGF-β/SMAD3 signaling modulation (Kanasaki et al., 2014; Shi et al., 2015; Shi et al., 2016). Here, we analyzed TGF-β/SMAD3 signaling *in vivo* and vitro. Western blot analysis revealed that the expression of TGFβR1, TGFβR2 and *p*-SMAD3 in the DM kidney were significantly induced when compared with control mice; however, expression was restored to control levels when DM were treated with H19 shRNA (Figures 4A–D), suggesting that STZ-induced TGF-β/SMAD3 signaling was suppressed by H19 knockdown. TGF-β2 induced TGF-β/SMAD3 was similarly suppressed by H19 knockdown in HMVECs (Figures 4E–H). Furthermore, wound healing cell invasion assays revealed that TGF-β2 induced the migration of HMVECs, while H19 knockdown inhibited their invasion (Figures 4I,J). The Boyden chamber cell migration assay also revealed that H19 knockdown inhibited endothelial cell transmigration through Matrigel (Figures 4K,L). These data reveal that TGF-β/SMAD3 signaling may be the key pathway in the protective role for kidney fibrosis in H19 knockdown in DM.

## Discussion

In this research, our preliminarily data confirmed that H19 expression was significantly up-regulated in TGF-β2-induced HMVEC fibrosis and in the fibrotic kidneys of STZ induced diabetic CD-1 mice. H19 knockdown significantly attenuated kidney fibrosis *in vitro* and *in vivo*, which was associated with the inhibition of the EndMT associated FSP-1. We also found that the up-regulated H19 observed in diabetic kidneys may be associated with suppressed levels of miR-29a in DM. H19, miR-29a, and EndMT contribute to a regulatory network involved in kidney fibrosis, all of which were associated with the regulation of the TGF-β/SMAD3 singling pathway.

LncRNA, initially thought to be transcriptional noise, have been intensely studied in recent years and they have been found to participate in gene expression, mammalian development, and various disease processes (Ponting et al., 2009; Caley et al., 2010). Several lines of evidence indicate that lncRNAs are responsible for renal cell apoptosis in DN (Kato et al., 2016; Long et al., 2016; Chen et al., 2017; Tsai et al., 2018). Recent evidence demonstrates that lncRNAs also mediate renal fibrosis in DN, such as the lncRNA NEAT1 and lncRNA ASncmtRNA-2 which induce kidney fibrosis in DN, and 1700020I14Rik and lncRNAGm4419 which attenuate kidney fibrosis in DN (Gao et al., 2017; Yi et al., 2017; Huang et al., 2019). In this study, we found that H19 knockdown can attenuate kidney fibrosis *in vivo* and *in vitro*. Xie et al. (2016) also found that H19, along with miR-17 and fibronectin, contributed to a regulatory network involved in renal fibrosis.

Inhibition of kidney fibrosis is a fundamental process in research on developing therapies against kidney disease, although kidney fibroblasts have been implicated in kidney fibrosis pathogenesis, inoculating only kidney fibroblasts as therapeutic targets would be challenging. The inhibition of kidney fibrosis and the restoration of normal kidney structure are fundamental processes to combat the progression of DN. Our previous research found that EndMT is very important in the progression of kidney fibrosis (Kanasaki et al., 2014). In our analysis, the expression of H19 was induced in the diabetic kidney in a time dependent manner and was associated with the progress and severity of kidney fibrosis. H19 knockdown inhibited kidney fibrosis and restored normal kidney structure. These data confirmed that EndMT is a key factor in the progress of kidney fibrosis. We know that mRNA, miRNA, and lncRNAs can communicate with each other by competing for shared miRNA targets (Tay et al., 2014; Srivastava et al., 2016). To further examine the mechanism of H19 in fibrosis, we analyzed the miR-29 family members which have been shown to have an antifibrotic role in DN (Denzler et al., 2014; Kanasaki et al., 2014; Srivastava et al., 2016). We revealed that H19 knockdown can restore the suppressed miR-29a in the diabetic kidney and in TGF-β2-fibrosis-induced HMVECs. The TGF-β/SMAD signaling pathway being key pathway to both. Thus, H19, miR-29a, and EndMT contribute to a competing endogenous RNA regulatory network. This regulatory network maintained a relative balance to avoid abnormal kidney fibrosis. When H19 was induced in kidney fibrosis, elevated H19 expression could alleviate the repressive effects of miR-29a and lead to increased EndMT associated gene expression, which is a target gene of miRNA-29 family. Similar H19 regulatory mechanisms have previously been reported such as the finding that the H19/miR-675 pathway inhibited cell growth and Igf1r expression (Keniry et al., 2012); H19/Let-7-mediated inhibition on the target HMGA2-mediated epithelial to mesenchymal transition (Ma et al., 2014); and the H19/miR-675 axis inhibits prostate cancer metastasis via affecting TGF-β1 expression (Zhu et al., 2014). Thus, H19 may act as a competitive endogenous RNA. The regulatory network integrates the transcriptional and posttranscriptional regulatory network of kidney fibrosis.

**FIGURE 5:**
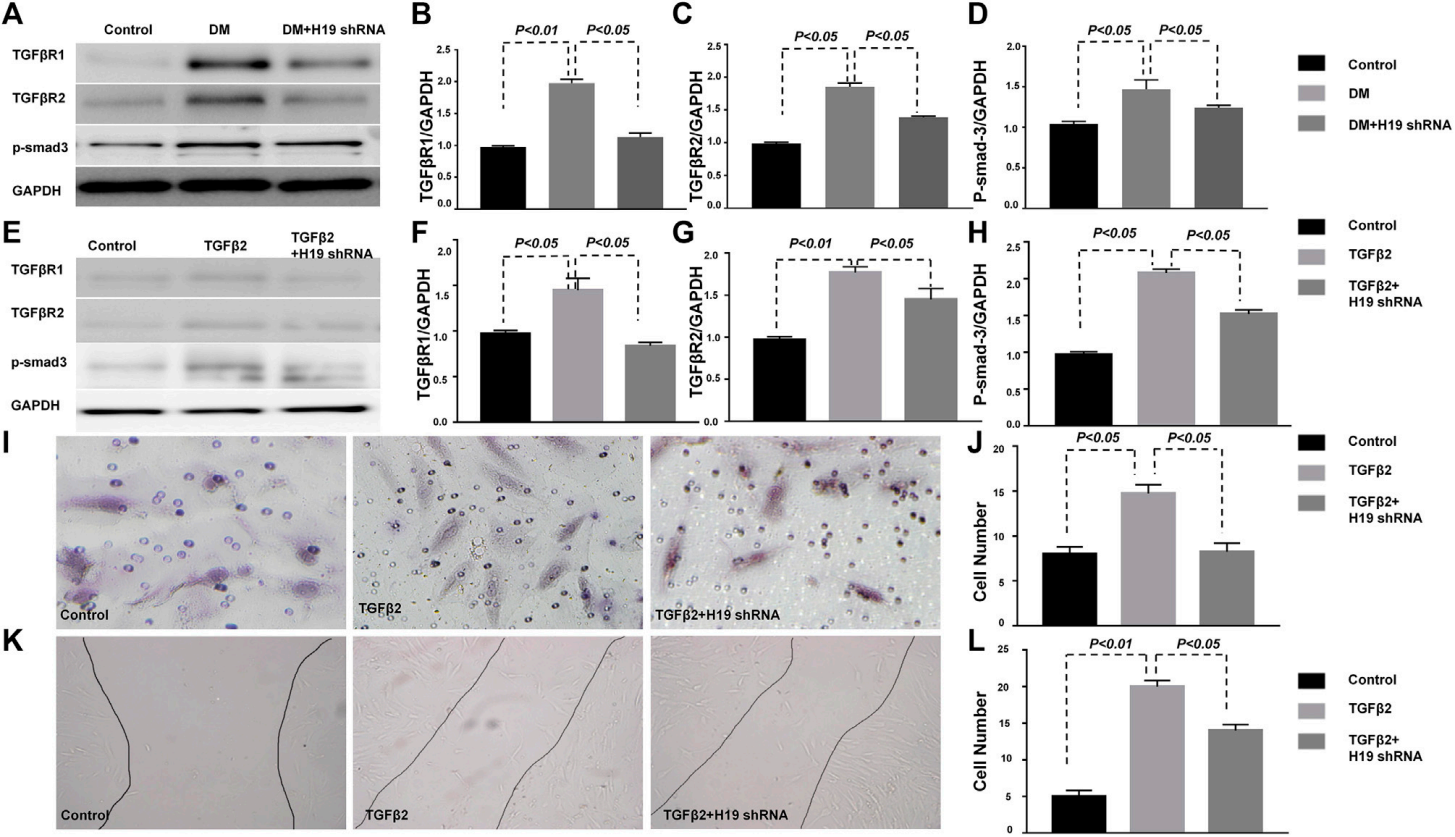
TGF-*β*/SMAD signaling protects the kidney from fibrosis in H19 knockdown in HMVECs. **(A)**–**(D)** Western blot analysis of TGFβR1, TGFβR2, *p*-SMAD3 and GAPDH in the kidney; **(E)**–**(H)** Western blot analysis of TGFβR1, TGFβR2, *p*-SMAD3 and GAPDH in HMVECs; **(I)**–**(J)** Wound healing cell invasion assay; **(K)**–**(L)** The Boyden chamber cell migration assay. The data are presented as meanent in each group (n = 5) of three independent experiments.

In summary, our findings reveal high expression of the lncRNA H19 in the diabetic kidney and in TGF-β2 induced fibrosis in HMVECs. Inhibition of H19 attenuated kidney fibrosis and restored normal kidney structure (Figure 5). Interestingly, inhibition of H19 only altered miR-29a levels, not miR-29b or miR-29-c levels, inactived the TGF-β/SMAD pathway, in order to down-regulate EndMT, leading to the suppression of kidney fibrosis. All together our data suggest that suppression of H19 plays an anti-fibrotic role, which may serve as a novel therapeutic target for DN.

**FIGURE 6:**
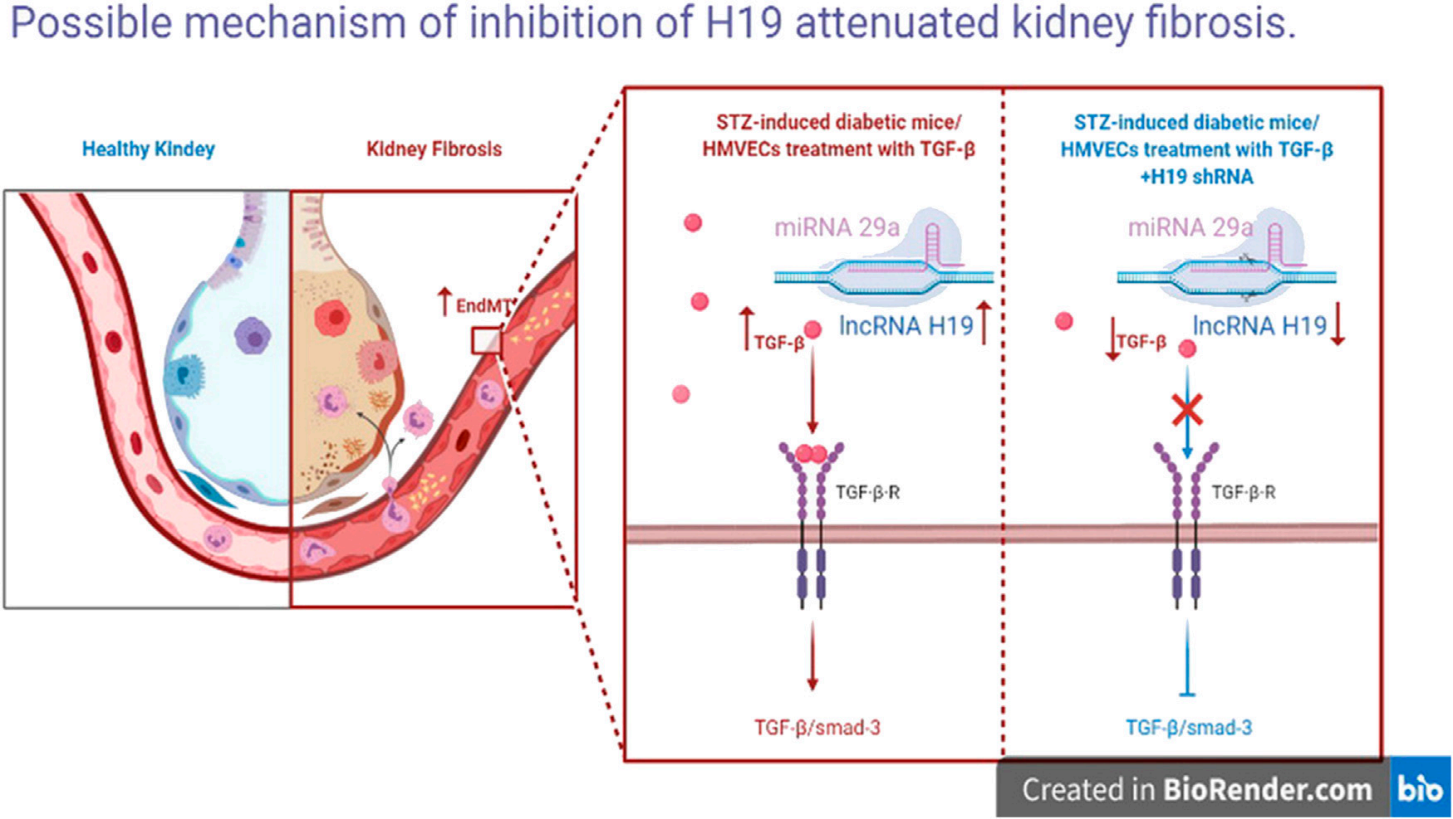
Possible mechanism of inhibition of H19 attenuated kidney fibrosis. Inhibition of H19 up-regulate miR-29a levels, inactive the TGF-GFSMAD pathway and down-regulate EndMT, leading to the suppression of kidney fibrosis.

## Data Availability Statement

The raw data supporting the conclusions of this article will be made available by the authors, without undue reservation.

## Ethics Statement

The animal study was reviewed and approved by the Ethics Committee of the affiliated Hospital of Southwest Medical University.

## Author Contributions

SS performed the research and wrote the paper. LS contributed important reagents and assisted in writing the paper. HY, SF, JH, and YL contributed to the animal experiments, and collected and analyzed data. YH designed the research project.

## Funding

This work was generously supported by grants from The National Natural Science Foundation of China (Grant No. 81500643).

## Conflict of Interest

The authors declare that the research was conducted in the absence of any commercial or financial relationships that could be construed as a potential conflict of interest.
